# ABO Blood Group System and Gastric Cancer: A Case-Control Study and Meta-Analysis

**DOI:** 10.3390/ijms131013308

**Published:** 2012-10-17

**Authors:** Zhiwei Wang, Lei Liu, Jun Ji, Jianian Zhang, Min Yan, Jun Zhang, Bingya Liu, Zhenggang Zhu, Yingyan Yu

**Affiliations:** Shanghai Key Laboratory of Gastric Neoplasms, Department of Surgery, Shanghai Institute of Digestive Surgery, Ruijin Hospital, Shanghai Jiao Tong University School of Medicine, Shanghai 200025, China; E-Mails: surgerywangzw@yahoo.com.cn (Z.W.); surgeryliul@yahoo.com.cn (L.L.); surgeryjij@yahoo.com.cn (J.J.); surgeryzhangjanian@yahoo.cn (J.Z.); surgeryyanm@yahoo.cn (M.Y.); surgeryzhangjun@yahoo.cn (J.Z.); surgeryliuby@yahoo.com.cn (B.L.); surgeryzhu@yahoo.com.cn (Z.Z.)

**Keywords:** ABO blood group, gastric cancer, *Helicobacter pylori*, Asian cohort, meta-analysis

## Abstract

This study focuses on the association between the ABO blood group system and the risk of gastric cancer or *Helicobacter pylori* infection. The data for the ABO blood group was collected from 1045 cases of gastric cancer, whereby the patient underwent a gastrectomy in Ruijin Hospital, Shanghai. The information on the ABO blood group from 53,026 healthy blood donors was enrolled as control. We searched the Pubmed database on the relationship between ABO blood groups and gastric cancer risk for meta-analysis. In our case-control study, the risk of gastric cancer in blood group A was significantly higher than that in non-A groups (O, B and AB) (odd ratio, OR1.34; 95% confidential interval, CI 1.25–1.44). Compared with non-O groups (A, B and AB), individuals with blood group O demonstrated a reduced risk of gastric cancer (OR = 0.80; 95% CI 0.72–0.88). The proportion of *H. pylori* infection in blood group A individuals was significantly higher than that in non-A blood groups (OR = 1.42; 95% CI 1.05–1.93). We further combined our data with the published data of others, and crossreferenced the risk of gastric cancer with the blood type, finding consistent evidence that gastric cancer risk in the blood A group was higher than that in the non-A groups (OR = 1.11; 95% CI 1.07–1.15), and that blood type O individuals were consistently shown gastric cancer risk reduction (OR = 0.91; 95% CI 0.89–0.94). Our study concluded that there was a slightly increased risk of gastric cancer in blood group A individuals, and people with blood type A are more prone to be infected by *H. pylori* than other ABO blood type individuals, whereas, a slightly decreased risk of gastric cancer was identified in blood type O individuals.

## 1. Introduction

Gastric cancer is the second most common cause of cancer death worldwide [1,2]. About one million patients are newly diagnosed with gastric cancer each year, with 700,000 deaths each year [1]. It is known that gastric cancer can be caused by the interaction between environmental factors and genetic variations [3–5]. As an environmental factor, Helicobacter pylori (*H. pylori*) infection plays an important role in the development of gastric cancer. However, only a small proportion of *H. pylori* carriers develop gastric cancers. Such clinical diversity indicates that there are likely to be other factors in gastric carcinogenesis, including genetic susceptibility of the host [6–8].

Aird *et al.* were the first to notice the correlation between gastric cancer and blood group A [9]. Since then, the relationship between ABO blood groups and carcinogenesis or progression of human tumors has been reported by many investigations, including increased breast cancer risk in blood group A [10]. In a hospital-based case-control study, increased pancreatic cancer risk among persons with blood type A [11], and increased ovarian cancer risk in women with blood group A than in others was reported [12]. However, the results regarding the relationship between blood group A and gastric cancer were inconsistent [9,13–15].

Meta-analysis is a well-established method that can pool the data from smaller inconclusive studies [16]. Compared to individual studies, the data yielded from meta-analysis disclosed greater statistical power and property for genetic risks analysis [17]. To understand the correlation between ABO blood groups and the risk of developing gastric cancer, we conducted a case-control study of 1,045 cases of gastric cancer from Shanghai Ruijin Hospital, and 53,026 healthy blood donors from Shanghai blood center. Moreover, we searched the Pubmed database and combined them with our own research data in order to perform an overall meta-analysis on the relationship between ABO blood groups and the gastric cancer risk. Current study provides a systematic knowledge on the correlation among human ABO blood groups, *H. pylori* susceptibility and gastric cancer risk.

## 2. Results

### 2.1. The Case-Control Study

A cohort case of 1,045 gastric cancer was collected from Shanghai Ruijin Hospital. Among them, 438 cases (41.91%) were blood group A, 233 cases (22.30%) group B, 289 cases (27.66%) group O and 85 cases (8.13%) group AB. As a control, a cohort of 53,026 healthy blood donors was collected from the local blood bank. Among them, 16,595 individuals (31.30%) were blood group A, 13,443 (25.35%) group B, 18,329 (34.57%) group O and 4,659 (8.78%) group AB. In comparing the gastric cancer group with the healthy controls, it becomes evident that the risk of gastric cancer in blood groups A was significantly higher than that in non-A groups (OR = 1.34, 95% CI 1.25–1.44). The individuals with blood group O showed a significant reduced risk of gastric cancer (OR = 0.80, 95% CI 0.72–0.88). The people with blood group B showed a significantly reduced risk of gastric cancer, relative to non-B group (OR = 0.88, 95% CI 0.78–0.99). The difference of gastric cancer risk between the blood group AB and the non-AB groups was not significant (OR = 0.93, 95% CI 0.75–1.14).

We compared the distribution of ABO blood groups in *H. pylori* positive cases and *H. pylori* negative cases in the gastric cancer group. In 67 cases of *H. pylori* positive gastric cancer patients, 35 cases (52.2%) were blood group A, 17 (25.4%) group B, 13 (19.4%) group O, and 2 (3.0%) group AB. By contrast, in 48 cases of *H. pylori* negative gastric cancer cases, 15 (31.3%) were blood group A, 11 (22.9%) group B, 16 (33.3%) group O, and 6 (12.5%) group AB. The ratio of *H. pylori* infection in blood group A cases was significantly higher than that in non-A blood group cases (OR = 1.42; 95% CI 1.05–1.93). Compared with non-O blood group, individuals with blood group O showed a reduced risk of *H. pylori* infection (OR = 0.71; 95% CI 0.46–1.10). People with blood group AB also showed a modestly reduced risk of *H. pylori* infection, compared with non-AB blood groups (OR = 0.41; 95% CI 0.12–1.38). The difference of *H. pylori* infection ratio between Blood group B and non-B blood groups was not significant (OR = 1.06; 95% CI 0.75–1.50).

### 2.2. Meta-Analysis

**Figure 1:**
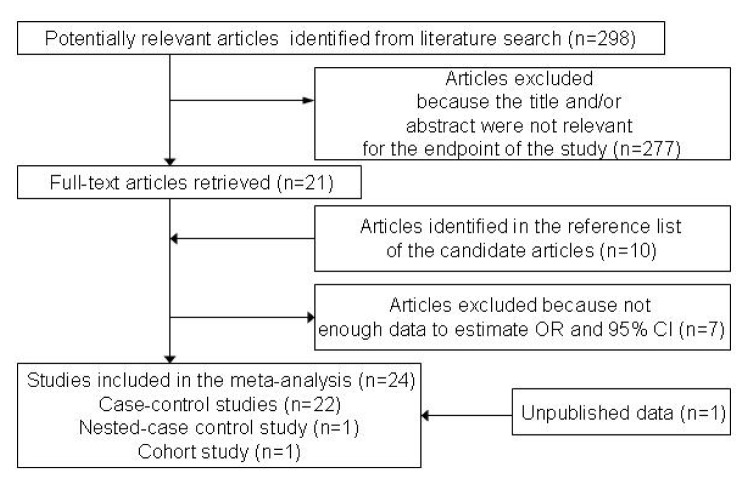
The flow diagram of the selection of studies.

**Figure 2:**
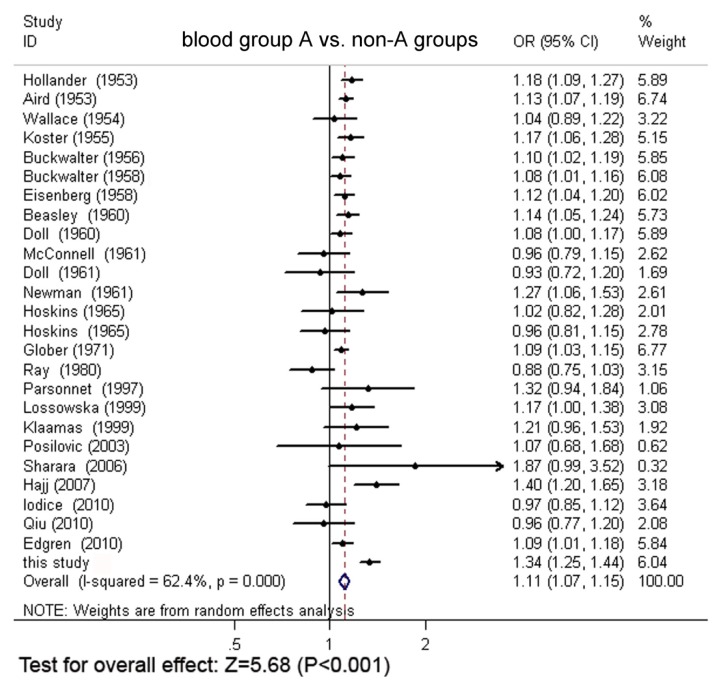
The forest plot for the relation of non-A groups *vs*. blood group A based on meta-analysis.

**Figure 5:**
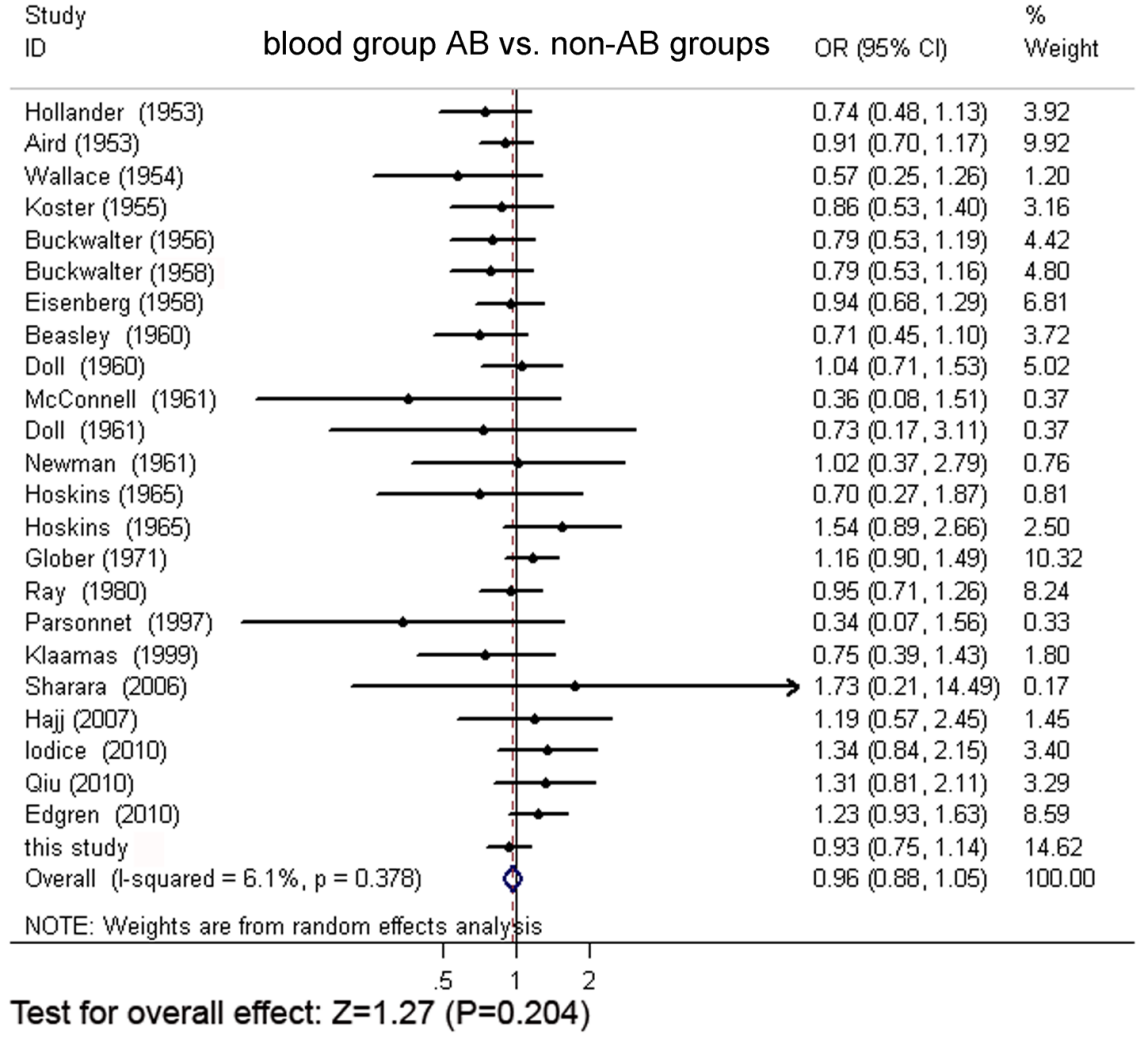
The forest plot for the relation of blood group AB and non-AB groups based on meta-analysis.

Through extensive research, a total of 24 original articles covered 15,843 gastric cancer cases and 1,421,740 controls were put into the meta-analysis. One of the articles contains two populations [18]. Meanwhile, our Asian cohort collected from Shanghai Ruijin Hospital was included in the meta-analysis (Figure 1, Table 1). The meta-analysis resulted in the following: the risk of gastric cancer in a blood group A individual was significantly higher than that in a non-A group (OR = 1.11, 95% CI 1.07–1.15), and the blood group O individuals showed a significant reduced risk of gastric cancer, compared with the non-O individuals (OR = 0.91, 95% CI 0.89–0.94). However, no significant difference was found when blood group B was compared with non-B groups (OR = 0.97, 95% CI 0.91–1.04) or when blood group AB was compared with non-AB groups (OR = 0.95, 95% CI 0.87–1.03). The forest plots for the relation between ABO blood groups and the risk of gastric cancer were presented in Figure 2 to Figure 5.

### 2.3. Meta-Regression Analyses

To evaluate the heterogeneity among the parameters, we performed meta-regression analysis on publication time, country (geographical factor), study design, and the proportion of ABO blood group individuals in cases and controls (sample constitution factors) (Table 2). For blood group B, the geographic factor (Coef. = 0.17, *p* = 0.015) and sample constitution factor (Coef. = 2.39, *p* = 0.001) were the reasons for heterogeneity. For blood group O, sample constitution factor (Coef. = 2.39, *p* = 0.001) was the source of heterogeneity (Coef. =1.14, *p* = 0.017).

Furthermore, we evaluated stratum-specific odds ratios, and the stratum-specific estimation for geographical region showed that OR for blood group B in Asian people is 1.141, 95% CI (1.065–1.122); in Europe, OR is 0.885, 95% CI (0.825–0.948); in America, OR is 0.981, 95% CI (0.887–1.085) and in mixed areas, OR is 0.928, 95% CI (0.757–1.138). Moreover, the overall OR for blood group B after stratum-specific analysis by geographical region is 0.978, 95% CI (0.929–1.029). The stratum-specific odds ratio for sample constitution in blood group B showed that, in Group One, OR is 4.822, 95% CI (4.505–5.160); while in Group Two, OR is 0.456, 95% CI (0.433–0.479). The overall OR for blood group B after stratum-specific analysis by sample constitution, then, is 0.978, 95% CI (0.929–1.029). Furthermore, the stratum-specific odds ratio for sample constitution in blood group O shows that, in Group One, OR is 6.443, 95% CI (6.315–6.575); in Group Two, OR is 0.265, 95% CI (0.254–0.276), and the overall OR for blood group O after stratum-specific analysis is 0.832, 95% CI (0.809–0.856).

### 2.4. Publication Bias

**Figure 6:**
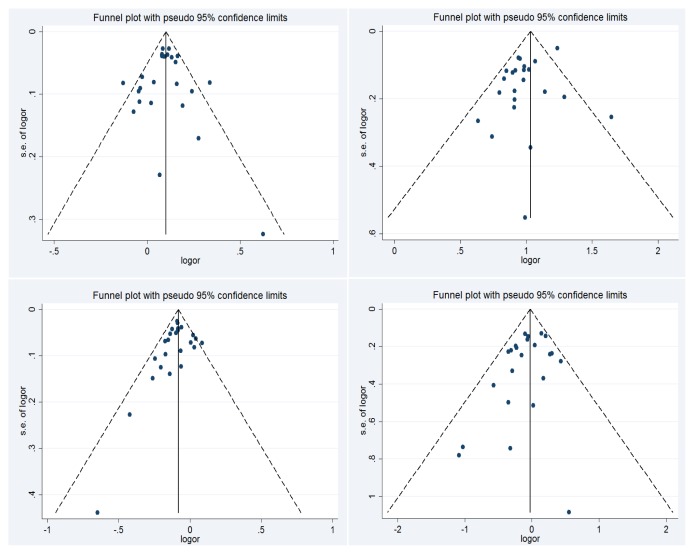
The Begg’s funnel plot analysis for blood group A *vs*. non-A groups and gastric cancer risk (**upper left**), blood group B *vs*. non-B groups (**upper right**), blood group O *vs*. non-O groups (**lower left**) and blood group AB *vs*. non-AB groups (**lower right**). The vertical axis represents the log of OR. The horizontal axis represents the SE of log (OR). The funnel plots are drawn with 95% confidence limits. OR: odds ratio; SE: standard error.

We used Begg’s test or Egger’s test to evaluate the publication bias. There were no significant publication bias for blood group A (Begg’s test, *p* = 0.83; Egger’s test, *p* = 0.77), blood group B (Begg’s test, *p* = 0.75; Egger’s test, *p* = 0.07), blood group O (Begg’s test, *p* = 0.08, Egger’s test, *p* = 0.27) and blood group AB (Begg’s test, *p* = 0.21; Egger’s test, *p* = 0.14). The Begg’s funnel plot for the relation between ABO blood groups and the risk of gastric cancer are shown in Figure 6.

## 3. Discussion

The relationship between the ABO blood group system and the incidence of tumor has been noticed for many years. Recently, Iodice *et al.* reported that the incidence risk of pancreatic cancer in blood group O individuals was significantly reduced compared with that in non-O group (RR = 0.79, 95% CI 0.70–0.90) in Europeans [19]. Meanwhile, they investigated the relationship between ABO blood groups and other kinds of cancer, but failed to confirm the relationship between ABO blood groups and gastric cancer. In our case-control study, we found a significant relationship between ABO blood groups and gastric cancer. The risk of gastric cancer in blood groups A was significantly higher than that in non-A groups.

A systematic meta-analysis helps researchers to summarize studies on specific topics. Since meta-analysis is a combination of multiple studies, it is less influenced by separate findings from a single study. In addition, meta-analyses reveal higher statistical power than traditional single studies. Regarding the effect of ABO blood groups on the risk of gastric cancer, we performed a meta-analysis combined with our case-control study from a large cohort. The meta-analysis indicated that the risk of gastric cancer in blood group A individuals was significantly higher than that in non-A group individuals (OR = 1.11, 95% CI 1.07–1.15), whereas the individuals with blood group O showed a significantly reduced risk of gastric cancer, compared with the non-O individuals (OR = 0.91, 95% CI 0.89–0.94). Odds ratios are commonly reported in the medical literature as the measure of association between exposure and outcome [20]. In this present study, the OR values of the relationship between blood group A and gastric cancer was in the range of 1.1–1.5, suggesting a weak association. The OR value of the relationship between blood group O and gastric cancer is in the range of 0.8–0.9, which also suggests a weak association. However, our meta-analysis failed to prove any relationship between blood group B or blood group AB with the gastric cancer risk. Regarding the heterogeneity of the publication language, data quality and data source, there are some limitations in meta-analysis. Therefore, the researchers need to search more articles including English articles and non-English articles in order to make their meta-analysis more reliable for guiding clinical work.

The association between blood group A and gastric cancer has been mentioned in the studies of several groups [9,13,14]. In regards to the possible explanations for the increased incidence of gastric cancer in blood group A individuals, Roberts *et al.* proposed that individuals with blood group A were more susceptible to pernicious anemia, compared with non-A blood group individuals [21]. A pernicious anemia individual is more prone to gastric cancer [18]. A clinical study demonstrated that altered gastric secretary function may be related to the ABO blood group. Compared to individuals with blood group A, the individuals of blood group O produced more free acid in their stomachs. The mean value of plasma pepsinogen in individuals with blood group O (564 units/mL) was higher than that in individuals with blood group A (494 units/mL) [22]. One study indicated that the immune-reaction to tumors in individuals with blood group A was reduced compared with the non-A blood group individuals [12,23]. Recently, one paper published in *Nature* journal indicated that the erythrocyte receptor-ligand pair is essential for erythrocyte invasion in the pathogenesis of malaria. They found that Ok blood group antigen, a kind of blood group antigen, is a receptor for erythrocyte invasion of the parasite [24]. This discovery suggested that individuals with the A blood group antigen may be more susceptible to *H. pylori* invasion. Therefore, we further analyzed the infection status in our gastric cancer cohort and found that the ratio of *H. pylori* infection in blood group A cases was significantly higher than that in non-A blood group cases. Recently, Nakao *et al*. analyzed the ABO genotype and the risk of gastric cancer, atrophic gastritis and *H. pylori* infection, and proposed that the risk for gastric cancer, atrophic gastritis and *H. pylori* infection was increased in AA genotype [25]. This finding is consistent with our data. *H. pylori* plays an important role in the development of gastric ulcer and gastric cancer. *H. pylori* is a Gram negative bacteria, which can be divided into cytotoxin-associated antigen (CagA) and vacuolating cytotoxin (VacA) positive strains, as well as CagA and VacA negative strains. CagA positive *H. pylori* suggest a considerably increased risk of gastric cancer [26]. The adhesion molecule system of *H. pylori* helps the bacteria colonizing in stomach mucosa. The most important adhesion molecular is blood group antigen-binding adhesion A (BabA). Adherence by *H. pylori* increases the risk of gastric disease. Animal experiments revealed that BabA could stimulate the inflammatory cells to release more interleukin-8, CCL5 proinflammatory cytokines and precancer-related factors (CDX2 and MUC2) [27]. Since inflammatory response to *H. pylori* infection plays an important role in cellular proliferation and gastric mucosal damage, the upregulation of proinflammatory cytokines in people with chronic gastritis may be an important clinical implication in gastric carcinogenesis.

## 4. Experimental Section

### 4.1. Case-Control Study

The ABO blood group information of 1,045 cases of hospitalized gastric cancer was from Shanghai Ruijin Hospital between 2002 and 2010, with age 25 to 90, median 60 years old. Among them, 709 cases are male and 336 cases are female. Of them, 115 cases obtained *H. pylori* infection information through 13C urea breath test before operation. The diagnosis of gastric cancer was confirmed histologically in all patients. The control group was composed of 53,026 healthy blood donors in 2010 from Shanghai central blood bank. Informed consent for participation in the study was obtained from all subjects with approval by the ethics committee of Ruijin Hospital.

Pearson’s chi-square test was used to compare the distribution of ABO blood groups between gastric cancer patients and controls. Odds ratios (OR) and 95% confidence intervals (CI) were used to evaluate the relationship between ABO blood groups and gastric cancer. All statistical analyses were performed with SPSS (Version 15.0; SPSS Inc.: Chicago, IL, USA). *p* < 0.05 indicated statistically significant differences.

### 4.2. Meta-Analysis and Statistics

We comprehensively searched the Pubmed database for all English literatures from 1953 to end of 2010. We combined the terms “stomach neoplasms [MeSH] OR gastric neoplasms [tiab] OR stomach cancer [tiab] OR gastric cancer[tiab] OR stomach carcinoma[tiab] OR gastric carcinoma [tiab] OR gastric tumor [tiab]” and “ABO blood group system [MeSH] OR Blood Group [tiab] OR Blood group antigens [tiab].” The selection of articles was accomplished independently by two researchers (Wang and Liu), and discrepancies were discussed with the third researcher (Yu), until consensus was reached. The literatures that meet the following criteria were included in the meta-analysis: (1) the study was either a case-control study, or a nested case-control study; (2) the study reported the definite numbers of individuals with blood groups A, B, O, and AB in gastric cancer cases and controls. Non-English articles, review articles, or articles which were involving the mechanism study were excluded from the study. We used a standardized protocol to extract the following data from each publication, including the first author, year of publication, study design, source of the control group, country in which the study was performed and the percentage of ABO blood groups in cases and controls (Table 1) [9,13,18,19,26,28–45].

Q and Higgins I^2^ statistics were used to examine heterogeneity among studies. In Q statistic, *p* value less than 0.05 means a heterogeneity. The extent of heterogeneity was evaluated by I^2^, which was documented as the percentage (*I*^2^ = 0%–25%, no heterogeneity; *I*^2^ = 25%–50%, moderate heterogeneity; *I*^2^ = 50%–75%, high heterogeneity; *I*^2^ = 75%–100%, extremely high heterogeneity) [46,47]. Fixed-effects model (Mantel Haenszel) or random-effects model (Dersimonian and Laird) was used depending on the heterogeneity between studies. The fixed-effects model was used if there was no significant heterogeneity, otherwise, the random-effects model was used [46]. To estimate the one or more covariates related to heterogeneity, meta-regression, as an extension to random-effects meta-analysis, was employed. Publication time is a continuous variable. Whole data are divided into two case-control studies and other studies. Geographical region is divided into Asia, Europe, America and other areas. As for a sample constitution, we first computed the mean of the proportion of people with different ABO blood groups in all individuals. The proportion of persons with different ABO blood groups was higher than the mean that was deemed as Group One, while the proportion of individuals with different ABO blood groups was lower than the mean that was deemed as Group Two. The overall effect was assessed by *Z* test. Funnel plots and Egger’s regression asymmetry test or Begg’s test were used to probe for publication bias [48,49]. Forest plots were used for graphic display. A *p* value of <0.05 was considered statistically significant. All data were analyzed with STATA (Version 11.0; Stata Corp.: College Station, TX, USA).

## 5. Conclusions

In conclusion, the present study confirmed that gastric cancer risk is increased in individuals with blood group A, as opposed to those in non-A blood groups. The individuals with blood group O showed a significant reduced risk of gastric cancer comparing with non-O blood groups. The susceptibility of blood group A individuals to gastric cancer may be partially attributed to an increased risk of *H. pylori* infection. This study implied that there must be a host genetic susceptibility for gastric cancer. However, the exact molecular mechanism underlying the relationship between ABO blood groups, *H. pylori* infection and gastric cancer needs to be further explored.

## Figures and Tables

**Figure 3:**
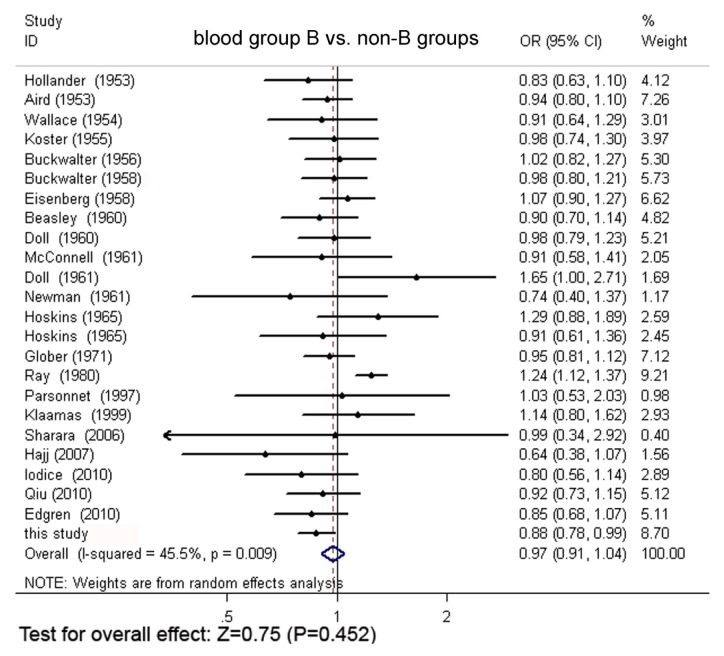
The forest plot for the relation of blood group B and non-B group based on meta-analysis.

**Figure 4:**
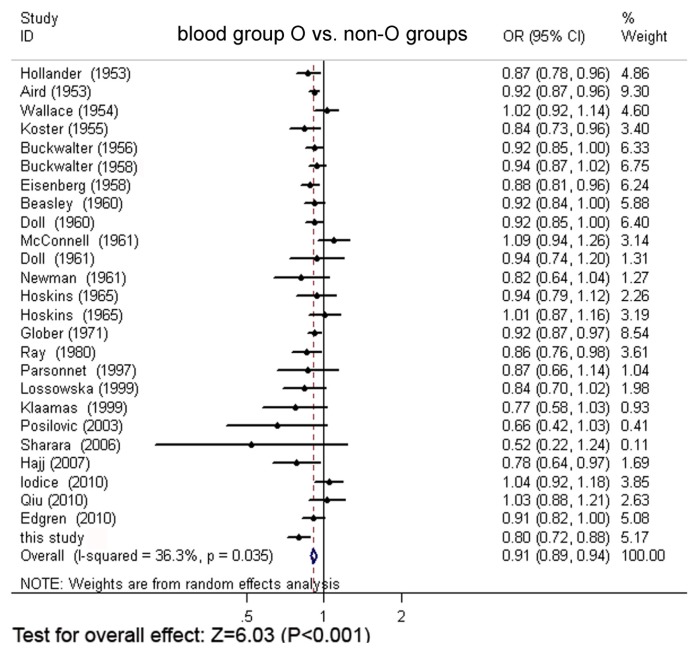
The forest plot for the relation of blood group O and non-O group based on meta-analysis.

**Table 1:** The literature about ABO blood groups and gastric cancer included in the meta-analysis.

First author	Publication year	Study type	Source of controls	Reference	Gastric cancer					Healthy control				
	
					Cases No.	Type A (%)	Type B (%)	Type O (%)	Type AB (%)	Controls No.	Type A (%)	Type B (%)	Type O (%)	Type AB (%)
Hollander *	1953	NA	NA	22	704	53.1	7.5	36.2	3.12	4518	45.1	9	41.7	4.25
Aird	1953	CC	Hospital/voluntary donors	9	3632	44.8	7.8	44.5	2.89	3632	39.8	8.3	48.6	3.19
Wallace	1954	CC	Voluntary donors	23	299	34.8	10	53.2	2	7418	33.5	11	51.9	3.55
Koster	1955	CC	Voluntary donors	24	413	51.3	11	34.1	3.87	14304	44	11	40.6	4.5
Buckwalter	1956	CC	Voluntary donors	27	879	45.9	9.2	42.1	2.84	6313	41.6	9	45.8	3.58
Buckwalter	1958	CC	Voluntary donors	25	908	45.8	9.3	42.2	2.75	49979	42.3	9.4	44.8	3.5
Eisenberg	1958	CC	Voluntary donors	29	892	45	12	38.6	4.04	75904	40.3	12	43.7	4.3
Beasley	1960	CC	NA	13	746	46.9	8.7	41.7	2.68	8084	41.1	9.7	45.4	3.8
Doll	1960	CC	Hospital	30	857	45.7	8.8	42.2	3.26	10000	42.2	8.9	45.8	3.13
Doll	1961	CC	Hospital/voluntary donors	34	229	37.6	9.6	52	0.87	860	39.3	11	47.7	2.44
McConnell	1961	NA	NA	36	105	40	16	41.9	1.9	610	43	9.8	44.6	2.62
Newman	1961	CC	Hospital/voluntary donors	31	118	51.7	8.5	36.4	3.39	1261	40.6	11	44.7	3.33
Hoskins	1965	CC	Voluntary donors	26	146	34.9	16	46.6	2.74	5236	34.2	12	49.7	3.9
Hoskins	1965	CC	Hospital/voluntary donors	26	223	36.3	10	47.5	5.83	4222	37.7	11	47.2	3.79
Glober	1971	CC	Hospital/voluntary donors	37	1680	45.9	8.5	42	3.57	43026	42.2	8.9	45.8	3.09
Ray	1980	CC	Voluntary donors	35	584	21.1	42	29.1	7.71	8821	24	34	33.9	8.15
Parsonnet	1997	NCC	cohort members	38	103	41.8	13	43.7	1.94	139	31.7	12	50.4	5.76
Lossowska	1999	CC	Voluntary donors	39	417	42.9	NA	30.2	NA	454	36.6	NA	35.9	NA
Klaamas	1999	CC	Voluntary donors	32	171	42.7	23	26.9	7.02	298	35.2	21	34.9	9.4
Posilović	2003	CC	Hospital	33	62	37.1	NA	30.7	NA	75	34.7	NA	46.7	NA
Sharara	2006	CC	Voluntary donors	40	15	46.7	20	26.7	6.67	104	25	20	51	3.85
Hajj	2007	CC	Hospital/voluntary donors	41	152	50	8.6	36.8	4.61	18972	35.6	14	47	3.88
Iodice	2010	CC	Hospital	42	301	39.2	9.3	45.9	5.65	15058	40.2	12	43.9	4.2
Qiu	2010	CC	Hospital	43	474	26.2	24	41.4	8.44	404	27.2	26	40.1	6.44
Edgren	2010	Cohort study	Voluntary donors	28	688	48.1	9.6	35.8	6.54	1089022	44	11	39.4	5.31
this study		CC	Voluntary donors		1045	41.9	22	27.7	8.13	53026	31.3	25	34.6	8.79

* Note: The data was cited in Aird’s article; Abbreviations: not available (NA); case-control study (CC); nested case-control study (NCC).

**Table 2:** Meta-regression analysis of ABO blood groups with covariates of interest.

Variable	Blood group A				Blood group B				Blood group O			
		
	Coef.	95% CI	*t*	*P* value	Coef.	95% CI	*t*	*P* value	Coef.	95% CI	*t*	*P* value
Publication year	0.001	(−0.001,0.002)	0.55	0.586	−0.003	(−0.01,0.001)	−1.97	0.067	0.001	(−0.001,0.002)	1.29	0.261
Area	−0.03	(−0.09,0.03)	−1.09	0.292	0.17	(0.03,0.30)	2.74	**0.015**	−0.01	(0.06,0.04)	−0.4	0.693
Study type	0.08	(−0.04,0.20)	1.4	0.181	−0.21	(−0.48,0.06)	−1.64	0.122	−0.03	(−0.15,0.08)	−0.61	0.548
Source of control	−0.01	(−0.06,0.05)	−0.28	0.785	0.08	(−0.04,0.20)	1.35	0.198	−0.01	(−0.06,0.04)	−0.32	0.755
Sample constitution	0.88	(−0.06,1.83)	1.97	0.066	2.39	(1.11,3.66)	3.99	**0.001**	1.14	(0.23,2.06)	2.65	**0.017**

Abbreviations: Coef., coefficient; CI, confidence interval.

## References

[1] Parkin D.M., Bray F., Ferlay J., Pisani P. (2005). Global cancer statistics, 2002. CA Cancer J. Clin.

[2] Brenner H., Rothenbacher D., Arndt V. (2009). Epidemiology of stomach cancer. Methods Mol. Biol.

[3] Tsugane S. (2005). Salt, salted food intake, and risk of gastric cancer: epidemiologic evidence. Cancer Sci.

[4] Murakami K., Kodama M., Fujioka T. (2006). Latest insights into the effects of *Helicobacter pylori* infection on gastric carcinogenesis. World J. Gastroenterol.

[5] Kelley J.R., Duggan J.M. (2003). Gastric cancer epidemiology and risk factors. J. Clin. Epidemiol.

[6] El-Omar E.M., Carrington M., Chow W.H., McColl K.E., Bream J.H., Young H.A., Herrera J., Lissowska J., Yuan C.C., Rothman N. (2000). Interleukin-1 polymorphisms associated with increased risk of gastric cancer. Nature.

[7] Gonzalez C.A., Sala N., Capella G. (2002). Genetic susceptibility and gastric cancer risk. Int. J. Cancer.

[8] Silva F., Carvalho F., Peixoto A., Seixas M., Almeida R., Carneiro F., Mesquita P., Figueiredo C., Nogueira C., Swallow D.M. (2001). MUC1 gene polymorphism in the gastric carcinogenesis pathway. Eur. J. Hum. Genet.

[9] Aird I., Bentall H.H., Roberts J.A. (1953). A relationship between cancer of stomach and the ABO blood groups. Br. Med. J.

[10] Hems G. (1970). Epidemiological characteristics of breast cancer in middle and late age. Br. J. Cancer.

[11] Vioque J., Walker A.M. (1991). Pancreatic cancer and ABO blood types: a study of cases and controls. Med. Clin.

[12] Henderson J., Seagroatt V., Goldacre M. (1993). Ovarian cancer and ABO blood groups. J. Epidemiol. Community Health.

[13] Beasley W.H. (1960). Blood groups of gastric ulcer and carcinoma. Br. Med. J.

[14] Roberts J.A. (1959). Some associations between blood groups and disease. Br. Med. Bull.

[15] Wiener A.S. (1962). Blood-groups and disease. A critical review. Lancet.

[16] Casas J.P., Hingorani A.D., Bautista L.E., Sharma P. (2004). Meta-analysis of genetic studies in ischemic stroke: Thirty-two genes involving approximately 18,000 cases and 58,000 controls. Arch. Neurol.

[17] Banerjee I., Gupta V., Ganesh S. (2007). Association of gene polymorphism with genetic susceptibility to stroke in Asian populations: a meta-analysis. J. Hum. Genet.

[18] Hoskins L.C., Loux H.A., Britten A., Zamcheck N. (1965). Distribution of ABO blood groups in patients with pernicious anemia, gastric carcinoma and gastric carcinoma associated with pernicious anemia. N. Engl. J. Med.

[19] Iodice S., Maisonneuve P., Botteri E., Sandri M.T., Lowenfels A.B. (2010). ABO blood group and cancer. Eur. J. Cancer.

[20] Viera A.J. (2008). Odds Ratios and Risk Ratios: What’s the Difference and Why Does It Matter?. South. Med. J.

[21] Roberts J.A. (1957). Blood groups and susceptibility to disease: A review. Br. J. Prev. Soc. Med.

[22] Sievers M.L. (1959). Hereditary aspects of gastric secretory function; race and ABO blood groups in relationship to acid and pepsin production. Am. J. Med.

[23] Smith D.F., Prieto P.A., Roitt I.M., Delves P.J. (1992). Forssmann Antigen. Encyclopedia of Immunology.

[24] Crosnier C., Bustamante L.Y., Bartholdson S.J., Bei A.K., Theron M., Uchikawa M., Mboup S., Ndir O., Kwiatkowski D.P., Duraisingh M.T. (2011). Basigin is a receptor essential for erythrocyte invasion by *Plasmodium falciparum*. Nature.

[25] Nakao M., Matsuo K., Ito H., Shitara K., Hosono S., Watanabe M., Ito S., Sawaki A., Iida S., Sato S. (2011). ABO genotype and the risk of gastric cancer, atrophic gastritis, and Helicobacter pylori infection. Cancer Epidemiol. Biomark. Prev.

[26] Parsonnet J., Friedman G.D., Orentreich N., Vogelman H. (1997). Risk for gastric cancer in people with CagA positive or CagA negative Helicobacter pylori infection. Gut.

[27] Ishijima N., Suzuki M., Ashida H., Ichikawa Y., Kanegae Y., Saito I., Boren T., Haas R., Sasakawa C., Mimuro H. (2011). BabA-mediated adherence is a potentiator of the *Helicobacter pylori* type IV secretion system activity. J. Biol. Chem.

[28] Wallace J. (1954). Blood groups and disease. Br. Med. J.

[29] Koster K.H., Sindrup E., Seele V. (1955). ABO blood-groups and gastric acidity. Lancet.

[30] Buckwalter J.A., Knowler L.A. (1958). Blood donor controls for blood group disease researches. Am. J. Hum. Genet.

[31] Buckwalter J.A., Wohlwend E.B., Colter D.C., Tidrick R.T. (1956). Natural selection associated with the ABO blood group. Science.

[32] Edgren G., Hjalgrim H., Rostgaard K., Norda R., Wikman A., Melbye M., Nyren O. (2010). Risk of gastric cancer and peptic ulcers in relation to ABO blood type: A cohort study. Am. J. Epidemiol.

[33] Eisenberg H., Greenberg R.A., Yesner R. (1958). ABO blood groups and gastric cancer. J. Chronic. Dis.

[34] Doll R., Swynnerton B.F., Newell A.C. (1960). Observations on blood group distribution in peptic ulcer and gastric cancer. Gut.

[35] Newman E., Naifeh G.S., Auer J.E., Buckwalter J.A. (1961). Secretion of ABH antigens in peptic ulceration and gastric carcinoma. Br. Med. J.

[36] Klaamas K., Kurtenkov O., Covacci A., Lipping A., Wadstrom T. (1999). Immune response to a recombinant fragment of the CagA protein of Helicobacter pylori in blood donors and patients with gastric cancer: Relation to ABO(H) blood group phenotype, stage of the disease and tumor morphology. Med. Microbiol. Immunol.

[37] Zivanovic-Posilovic G., Milicic J., Bozicevic D. (2003). Dermatoglyphs and gastric cancer. Coll. Antropol.

[38] Doll R., Drane H., Newell A.C. (1961). Secretion of blood group substances in duodenal, gastric and stomal ulcer, gastric carcinoma, and diabetes mellitus. Gut.

[39] Ray A.K. (1980). Blood Groups and Cancer in India. Curr. Anthropol.

[40] McConnell R.B. (1961). The mechanism by which blood groups antigens influence gastrointestinal disorders. Gastroenterology.

[41] Glober G.A., Cantrell E.G., Doll R., Peto R. (1971). Interaction between ABO and rhesus blood groups, the site of origin of gastric cancers, and the age and sex of the patient. Gut.

[42] Lissowska J., Groves F.D., Sobin L.H., Fraumeni J.F., Nasierowska-Guttmejer A., Radziszewski J., Regula J., Hsing A.W., Zatonski W., Blot W.J. (1999). Family history and risk of stomach cancer in Warsaw, Poland. Eur. J. Cancer Prev.

[43] Sharara A.I., Abdul-Baki H., ElHajj I., Kreidieh N., Kfoury Baz E.M. (2006). Association of gastroduodenal disease phenotype with ABO blood group and *Helicobacter pylori* virulence-specific serotypes. Dig. Liver Dis.

[44] El H., Hashash J.G., Baz E.M., Abdul-Baki H., Sharara A.I. (2007). ABO blood group and gastric cancer: rekindling an old fire?. South. Med. J.

[45] Qiu M.Z., Zhang D.S., Ruan D.Y., Luo H.Y., Wang Z.Q., Zhou Z.W., Wang F.H., Li Y.H., Xu R.H. (2011). A relationship between ABO blood groups and clinicopathologic characteristics of patients with gastric adenocarcinoma in China. Med. Oncol.

[46] DerSimonian R., Laird N. (1986). Meta-analysis in clinical trials. Control. Clin. Trials.

[47] Higgins J.P., Thompson S.G., Deeks J.J., Altman D.G. (2003). Measuring inconsistency in meta-analyses. BMJ.

[48] Thornton A., Lee P. (2000). Publication bias in meta-analysis: its causes and consequences. J. Clin. Epidemiol.

[49] Egger M., Smith G.D., Schneider M., Minder C. (1997). Bias in meta-analysis detected by a simple, graphical test. BMJ.

